# The likelihood of severe COVID‐19 outcomes among PLHIV with various comorbidities: a comparative frequentist and Bayesian meta‐analysis approach

**DOI:** 10.1002/jia2.25841

**Published:** 2021-11-19

**Authors:** Haoyi Wang, Kai J. Jonas

**Affiliations:** ^1^ Department of Work and Social Psychology Maastricht University Maastricht the Netherlands

**Keywords:** comorbidities, corona virus, COVID‐19, HIV, meta‐analysis

## Abstract

**Introduction:**

The SARS‐CoV‐2 virus can currently pose a serious health threat and can lead to severe COVID‐19 outcomes, especially for populations suffering from comorbidities. Currently, the data available on the risk for severe COVID‐19 outcomes due to an HIV infection with or without comorbidities paint a heterogenous picture. In this meta‐analysis, we summarized the likelihood for severe COVID‐19 outcomes among people living with HIV (PLHIV) with or without comorbidities.

**Methods:**

Following PRISMA guidelines, we utilized PubMed, Web of Science and medRxiv to search for studies describing COVID‐19 outcomes in PLHIV with or without comorbidities up to 25 June 2021. Consequently, we conducted two meta‐analyses, based on a classic frequentist and Bayesian perspective of higher quality studies.

**Results and discussion:**

We identified 2580 studies (search period: January 2020–25 June 2021, data extraction period: 1 January 2021–25 June 2021) and included nine in the meta‐analysis. Based on the frequentist meta‐analytical model, PLHIV with diabetes had a seven times higher risk of severe COVID‐19 outcomes (odd ratio, OR = 6.69, 95% CI: 3.03–19.30), PLHIV with hypertension a four times higher risk (OR = 4.14, 95% CI: 2.12–8.17), PLHIV with cardiovascular disease an odds ratio of 4.75 (95% CI: 1.89–11.94), PLHIV with respiratory disease an odds ratio of 3.67 (95% CI: 1.79–7.54) and PLHIV with chronic kidney disease an OR of 9.02 (95% CI: 2.53–32.14) compared to PLHIV without comorbidities. Both meta‐analytic models converged, thereby providing robust summative evidence. The Bayesian meta‐analysis produced similar effects overall, with the exclusion of PLHIV with respiratory diseases who showed a non‐significant higher risk to develop severe COVID‐19 outcomes compared to PLHIV without comorbidities.

**Conclusions:**

Our meta‐analyses show that people with HIV, PLHIV with coexisting diabetes, hypertension, cardiovascular disease, respiratory disease and chronic kidney disease are at a higher likelihood of developing severe COVID‐19 outcomes. Bayesian analysis helped to estimate small sample biases and provided predictive likelihoods. Clinical practice should take these risks due to comorbidities into account and not only focus on the HIV status alone, vaccination priorities should be adjusted accordingly.

## 1 INTRODUCTION

The ongoing severe acute respiratory syndrome coronavirus 2 (SARS‐CoV‐2, hereafter COVID‐19) pandemic renders an assessment of the risk of developing severe COVID‐19‐related outcomes among people living with HIV (PLHIV) and comorbidities in comparison to PLHIV only (HIV monoinfection) of utmost importance. Three meta‐analyses and systematic reviews on risk factors of acquiring severe COVID‐19 suggested an increased risk of severity of COVID‐19 among people living with diabetes, hypertension (HPT), cardiovascular disease (CVD) and respiratory disease (RD) [1, 2, 3]. This is also reflected in national health protection agency guidelines, for example CDC lists individuals with these comorbidities as an at‐risk population [4]. Data on the influence of an HIV infection on the development of severe COVID‐19 outcomes are, however, inconsistent. Earlier reviews on the interplay between HIV and COVID‐19 summarized that most of the case reports and prospective cohort studies suggested a similar risk of COVID‐19 severe outcome development compared to HIV‐negative individuals [5, 6] and individual studies point towards the importance of comorbidities (e.g. HPT [6]) and psychosocial factors [7]. At the same time, there is evidence for an increased risk. First of all, a small case series in Wuhan observed a higher proportion of severe cases among PLHIV compared to the general population. However, a large proportion of the sample in this study had discontinued HAART [8]. Second, two meta‐analyses concluded a moderately increased risk [9, 10]. So far, systematic review and meta‐analytical evidence on the direct link of HIV monoinfection and severe COVID‐19 progression can be judged as inconclusive. This inconsistency in risk assessment was confirmed by a systematic review on COVID‐19 outcomes in HIV/AIDS patients and two meta‐analyses on HIV and outcomes from COVID‐19 [11, 12], too.

The answer to these inconsistent findings most likely rests in the prevalence of comorbidities among PLHIV [13], especially HPT [6] and RDs [11]. The most prevalent age‐associated non‐communicable comorbidities (AANCCs) in PLHIV are HPT, CVD, RD/pulmonary disease and impaired renal function or other chronic kidney diseases (CKDs) [11, 14, 15, 16, 17], as well as diabetes [18, 19, 20, 21]. A first systematic review corroborates the assumptions regarding the role of AANCCs [22], but meta‐analytic evidence is still missing.

Therefore, to clarify the risks of severe COVID‐19 outcome among PLHIV with AANCCs, the meta‐analyses reported here aim to examine and quantify the risk of developing severe COVID‐19 related symptoms among individuals (mortality, hospitalization, severe and critical outcomes; see Statistical analysis section for a full description) living with HIV and AANCCs in comparison with individuals with HIV monoinfection. To account for a biased estimation of the meta‐analytic effect in frequentist meta‐analytic models with low amounts of studies entered, we also conducted a Bayesian meta‐analysis to compare parameters with the aim of improving the informational value of the analysis.

## 2 METHODS

### 2.1 Selection criteria and search strategy

This meta‐analysis is reported in accordance with the Preferred Reporting Items for Systematic Reviews and Meta‐Analyses (PRISMA) statement [23]. The PRISMA statement checklist report can be found in Appendix S1.

We selected relevant studies published between 1 January 2020 and 25 June 2021, by searching PubMed, Web of Science and medRxiv (data extraction period: 1 January 2021– 25 June 2021), using terms for HIV, relative comorbidities and COVID‐19 outcomes. Pertinent keywords and Medical Subject Headings (MeSH) terms related to these categories were used to maximize the output from the literature search (see Appendix S2 for full search terms). We also reviewed the references of included articles and other relevant systematic reviews and meta‐analysis to ensure the comprehensiveness of the research presented.

The inclusion criteria for this meta‐analysis were as following: (1) prospective original studies of COVID‐19 in PLHIV with reported number of AANCCs, such as diabetes, HPT, CVD, RD and CKD; (2) studies in human adult populations (18 years and older); (3) studies that reported the number of PLHIV only (monoinfection); and (4) pre‐print literature or other archived grey literature due to the emerging status of relevant studies. Studies which (1) were conducted among minors; (2) did not report severe COVID‐19 infections; (3) did not report the proportion of HIV monoinfections; and (4) case series, case reports, cross‐sectional studies, reviews or other systematic reviews/meta‐analysis, letters to the editor, opinion pieces, conference abstracts, dissertations/thesis and articles without the outcomes of interest were excluded from the screening process.

### 2.2 Data extraction and quality assessment

Data extraction and the evaluation of the literature quality were conducted by HW. Mendeley (version 1.19.4) was used to record all available information. The Newcastle–Ottawa Scale was used to assess the methodological quality of each study that met the selection criteria [24].

### 2.3 Statistical analysis

First, we extracted the absolute number of PLHIV reported by the selected studies for quantitative synthesis and grouped data by the type of HIV and comorbidities (diabetes, HPT, CVD, RD and CKD), and HIV monoinfection. We then collected information on the number of and the type of severe COVID‐19 outcome, which were defined as: mortality, hospitalization, severe and critical outcomes. Hospitalization was coded as a fact, and included the following outcomes: mortality; hospitalized but not requiring supplemental oxygen, hospitalized but requiring supplemental oxygen, hospitalized with non‐invasive ventilation and hospitalized on invasive mechanical ventilation. Severe outcome was defined as fever or suspected respiratory infection plus respiratory rate greater than 30 breaths per min, oxygen saturation of 93% or less on room air, or acute severe respiratory distress [acute lung infiltrate in chest imaging and ratio of partial pressure of arterial oxygen to fractional concentration of oxygen in inspired air (PaO_2_/FiO_2_) of ≤300]. Critical outcome was defined as rapid disease progression and respiratory failure with need for mechanical ventilation or organ failure that requires monitoring in an intensive care unit. Lastly, we re‐calculated the odds ratio (OR) of severe COVID‐19 outcomes between HIV monoinfections and HIV infections with the respective comorbidities through our meta‐analysis, instead of extracting the original relative risks, ORs or hazard ratios reported in the original studies.

#### 2.3.1 Classic frequentist meta‐analytical approach

In the classical frequentist approach, we used a random‐effects model and the DerSimonian–Laird method to estimate the model on a log‐OR scale. The DerSimonian–Laird *Q* test and *I* ^2^ values were used to assess heterogeneity, with low, moderate and high heterogeneity corresponding to *I* ^2^ values of 25%, 50% and 75%. In addition, heterogeneity τ was assessed in this study. We investigated a publication bias by inspecting funnel plots. To test the funnel plot asymmetry, we used both mixed‐effects meta‐regression model and rank correction test. The statistical analysis was carried out using R (version R 3.6.2) using the Metafor package v.2.4‐0 [25].

#### 2.3.2 Bayesian random‐effects meta‐analysis approach

To better estimate the between‐study variance in a context of limited numbers of studies to be included in the meta‐analysis, applying a Bayesian meta‐analysis increases the robustness of the model in the context of all sources of uncertainty, and incorporates external evidence on heterogeneity in the analysis [26]. Bayesian probability, contrary to the frequentist model, belongs to the category of evidential probabilities. It interprets probability as a reasonable expectation based on so‐called priors and compares them against so‐called posterior probabilities (evidence based). Choosing the adequate prior is thus essential in the Bayesian inference process. In sum, the Bayesian framework introduces a formal combination of a prior probability distribution (with a likelihood distribution of the pooled effect based on the observed data) to obtain a posterior probability distribution of the pooled effect [26]. An informative prior is necessary to precisely estimate heterogeneity [26]. Thus, we applied the half‐normal distribution with scale 0.5 as a prior for the analysis presented here, as this is recommended for log‐OR endpoints [27, 28]. Different prior distributions (half‐normal distribution with scale of 1.0 and half‐Cauchy distribution with scale of 0.5) were also applied for the purpose of the sensitivity analysis. Results were shown as the posterior distribution of the fixed effect μ on a log‐OR scale, heterogeneity τ and posterior knowledge of a “future” observation (prediction distribution). Both estimated fixed effects and random effects with 95% credible interval (CrI) were pitted against the estimates from the classical frequentist approach. The Bayesian meta‐analysis was carried out with the Bayesmeta package v.2.6 [28].

## 3 RESULTS AND DISCUSSION

### 3.1 Research selection and characteristics

**Figure 1:**
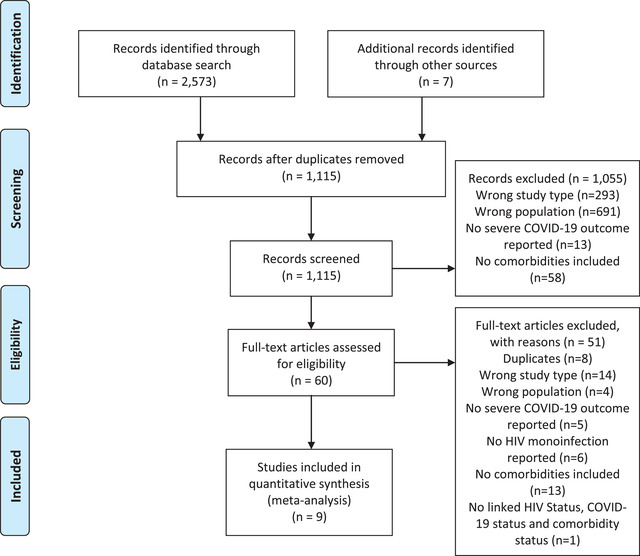
PRISMA flow diagram of the study selection process.

The search strategy identified 1115 studies after removing duplicates. We excluded 1055 studies after screening the titles and abstracts. Sixty studies remained for full‐text screening, after which 51 studies were excluded, leaving nine studies to be included in the meta‐analyses. Figure 1 shows the selection procedure in this study. Quality assessments are summarized in Table 1.

**Table 1:** Newcastle–Ottawa quality assessment of observational studies

Study, year	Study design	Selection	Comparability	Outcome	Total score	Result
Bhaskaran et al., 2021 [29]	Cohort study	***	**	**	7	Good
Boulle et al., 2020 [30]	Cohort study	***	**	**	7	Good
Ceballos et al., 2020 [31]	Cohort study	***	**	***	8	Good
Dandachi et al., 2020 [32]	Multi‐center registry	**	**	**	6	Fair
Etienne et al., 2020 [7]	Cohort study	***	**	**	7	Good
Isernia et al., 2020 [33]	Single‐center registry	*	**	**	5	Poor
Meyerowitz et al., 2020 [34]	Cohort study	***	**	**	7	Good
Pujari et al., 2021 [35]	Cohort study	***	**	**	7	Good
Vizcarra et al., 2020 [13]	Cohort study	****	**	**	8	Good

Note: The selection, comparability and exposure of each study were broadly assessed. Studies with 3 or 4 stars in selection domain AND 1 or 2 stars in comparability domain AND 2 or 3 stars in outcome/exposure domain were considered of good quality; studies with 2 stars in selection domain AND 1 or 2 stars in comparability domain AND 2 or 3 stars in outcome/exposure domain were considered of fair quality; or were considered as poor quality [24].

Of the selected studies (seven cohort studies and two registry studies), nine of them reported HIV and diabetes comorbidity; eight of them reported HIV plus HPT; six of them reported HIV plus CVD; six of them reported HIV plus RD; and five of them reported HIV plus CKD. In total, these studies included 32,037 individuals with an HIV/COVID‐19 co‐infection; among them 3248 were living with diabetes, 5478 living with HPT, 82 living with CVD, 67 living with RD and 1621 living with CKD (Table 2 and see Appendix S3 for the essential study characteristic details).

**Table 2:** Model summary

								Classical frequentist approach		Bayesian approach with half‐normal distribution prior^b^		
Comorbidity	Study	Year	Country	Sample size	COVID‐19 outcome	Log odds ratio (95% CI)	σ_i_	Pooled effect (95% CI)	Τ (95% CI)	Fixed effect (95% CrI)	Random effect τ (95% CrI)	Predictive distribution (95% CrI)
Diabetes	Bhaskaran et al. [29]	2021	UK	27,480	Mortality	3.63 (2.15–5.11)	0.57	1.90 (1.11–2.69)	0.82 (0.12–2.42)	1.92 (1.22–2.57)	0.54 (0–1.05)	1.94 (0.41–3.37)
	Boulle et al. [30]	2020	South Africa	3978	Mortality	2.26 (1.88–2.64)	0.04					
	Ceballos et al. [31]	2020	Chile	36	Mortality	2.2 (–0.65 to 5.04)	2.11					
	Dandachi et al. [32]	2020	US	286	Hospitalization^a^	1.14 (0.31–1.96)	0.18					
	Etienne et al. [7]	2020	France	54	Severe and critical	3.91 (0.84–6.98)	2.45					
	Isernia et al. [33]	2020	France	30	Hospitalization	2.93 (–0.16 to 6.03)	2.49					
	Meyerowitz et al. [34]	2020	US	36	Hospitalization	0.73 (–1.21 to 2.68)	0.98					
	Pujari et al. [35]	2021	India	86	Severe and critical	1.53 (–0.11 to 3.17)	0.70					
	Vizcarra et al. [13]	2020	Spain	51	Severe and critical	–0.47 (–2.86 to 1.92)	1.48					
Hypertension	Bhaskaran et al. [29]	2021	UK	27,480	Mortality	3.02 (1.55–4.50)	0.57	1.42 (0.75–2.10)	0.56 (0–1.84)	1.38 (0.76–2.02)	0.41 (0–0.97)	1.37 (0.20–2.62)
	Ceballos et al. [31]	2020	Chile	36	Mortality	2.2 (–0.42 to 4.81)	1.78					
	Dandachi et al. [32]	2020	US	286	Hospitalization^a^	1.08 (0.36–1.80)	0.13					
	Etienne et al. [7]	2020	France	54	Severe and critical	1.86 (0.40–3.32)	0.55					
	Isernia et al. [33]	2020	France	30	Hospitalization	2.25 (–0.84 to 5.34)	2.49					
	Meyerowitz et al. [34]	2020	US	36	Hospitalization	0.04 (–1.73 to 1.81)	0.82					
	Pujari et al. [35]	2021	India	86	Severe and critical	1.56 (0.16–2.96)	0.51					
	Vizcarra et al. [13]	2020	Spain	51	Severe and critical	0.37 (–1.14 to 1.88)	0.59					
Cardiovascular disease	Ceballos et al. [31]	2020	Chile	36	Mortality	3.3 (0.23–6.36)	2.44	1.56 (0.64–2.48)	0.58 (0–2.82)	1.55 (0.69–2.41)	0.35 (0–0.94)	1.55 (0.20–2.89)
	Dandachi et al. [32]	2020	US	286	Hospitalization^a^	1.95 (0.81–3.10)	0.34					
	Etienne et al. [7]	2020	France	54	Severe and critical	1.61 (0.27–2.95)	0.47					
	Isernia et al. [33]	2020	France	30	Hospitalization	1.84 ( –1.52 to 5.19)	2.94					
	Meyerowitz et al. [34]	2020	US	36	Hospitalization	2.4 (–0.78 to 5.57)	2.63					
	Vizcarra et al. [13]	2020	Spain	51	Severe and critical	–0.47(–2.33 to 1.39)	0.90					
Respiratory disease	Ceballos et al. [31]	2020	Chile	36	Mortality	0.75 (–2.89 to 4.38)	3.44	1.30 (0.58––2.02)	0 (0–1.43)	1.23 (0.35–2.08)	0.28 (0–0.82)	1.24 (–0.04 to 2.42)
	Dandachi et al. [32]	2020	US	286	Hospitalization^a^	1.58 (0.67–2.50)	0.22					
	Etienne et al. [7]	2020	France	54	Severe and critical	1.2 (–0.88 to 3.29)	1.13					
	Isernia et al. [33]	2020	France	30	Hospitalization	0.29 (–1.13 to 5.71)	3.04					
	Meyerowitz et al. [34]	2020	US	36	Hospitalization	0.22 (–2.14 to 2.58)	1.45					
	Vizcarra et al. [13]	2020	Spain	51	Severe and critical	0.22 (–2.29 to 2.74)	1.65					
Chronic kidney disease	Bhaskaran et al. [29]	2021	UK	27,480	Death	3.74 (2.20–5.27)	0.61	2.20 (0.93–3.47)	1.02 (0–3.96)	2.09 (1.15–3.07)	0.47 (0–1.08)	2.09 (0.54–3.72)
	Ceballos et al. [31]	2020	Chile	36	Mortality	2.2 (–0.65 to 5.04)	2.11					
	Dandachi et al. [32]	2020	US	286	Hospitalization^a^	1.58 (0.67–2.50)	0.22					
	Etienne et al. [7]	2020	France	54	Severe and critical	3.46 (0.33–6.60)	2.56					
	Meyerowitz et al. [34]	2020	US	36	Hospitalization	0.22 (–2.14 to 2.58)	1.45					

Note: Hospitalization includes hospitalized but not requiring supplemental oxygen, hospitalized but requiring supplemental oxygen, hospitalized with non‐invasive ventilation, hospitalized on invasive mechanical ventilation or ECMO and Mortality; Severe outcome was defined as fever or suspected respiratory infection plus respiratory rate greater than 30 breaths per min, oxygen saturation of 93% or less on room air or acute severe respiratory distress [acute lung infiltrate in chest imaging and ratio of partial pressure of arterial oxygen to fractional concentration of oxygen in inspired air (PaO2/FiO2) of ≤300]. Critically ill individuals were those with rapid disease progression and respiratory failure with need for mechanical ventilation or organ failure that needs monitoring in an intensive care unit.

^a^ Did not report type of hospitalization.

^b^ Half‐normal distributions with scale 0.5. 95% CI = 95% confident interval. 95% CrI = 95% credible interval. The scale on the effects is log odds ratio.

### 3.2 Association between HIV/comorbidity and risk of severe COVID‐19 outcomes

#### 3.2.1 HIV/diabetes comorbidity

**Figure 2:**
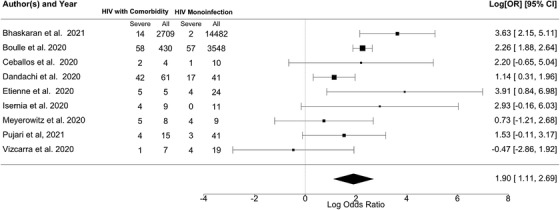
Forest plot of the association of HIV/diabetes in comparison to HIV monoinfection with severe COVID‐19 outcomes computed with classic frequentist approach. Note: Heterogeneity: *Q* = 18.35, *df* = 8, *p* = 0.0188, *I* ^2^ = 61.40%. The scale on the x‐axis is log odds ratio. Abbreviation: 95% CI, 95% confidence interval.

With the classical frequentist approach, compared to HIV monoinfection, the pooled odds estimate for PLHIV and diabetes comorbidity to develop severe COVID‐19 outcomes was higher [OR = 6.69, 95% confidence interval (CI) 3.03–19.30, τ = 0.82, 95% CI 0.12–2.42, *I* ^2^ = 61.40%, Figure 2].

**Figure 3:**
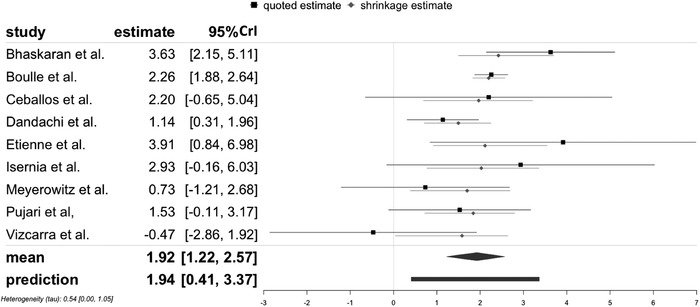
Forest plot of the association of HIV/diabetes in comparison to HIV monoinfection with severe COVID‐19 outcomes with Bayesian approach with half‐normal distribution prior (scale of 0.5). Note. The black line indicates the quoted estimates specified through effect from individual study and its σi as same as in the frequentist approach. The grey line presents the shrinkage intervals, which illustrate the posterior of each study's true effect. The black diamond presents the posterior distribution of the pooled effect, while the black bar shows the prediction distribution. The scale on the x‐axis is log odds ratio. Abbreviation: 95% CrI, 95% credible interval.

A similar but slightly larger fixed effect of the HIV/diabetes comorbidity compared to HIV monoinfection was estimated by the Bayesian approach with an OR of 6.82 (95% CrI 3.39–13.07, Figure 3). The random effect τ was estimated at 0.54 (95% CrI 0–1.05). The prediction distribution of the effect was estimated at a slightly higher value of 6.96 (95% CrI 1.51–29.08). All comparisons between two approaches are summarized in Table 2. Sensitivity analysis using different prior distributions can be found in Appendix S5. The sensitivity analysis showed that compared to the half‐normal distribution prior with scale of 0.5, results from the half‐normal distribution prior with scale 1.0 and the half‐Cauchy distribution prior with scale of 0.5 were more extreme. For consistent results in this meta‐analysis, all other models employing a Bayesian approach used half‐normal distribution prior with scale of 0.5. Detailed information for the posterior distributions can be found in Appendix S6.

#### 3.2.2 HIV/AANCC comorbidities

**Figure 4:**
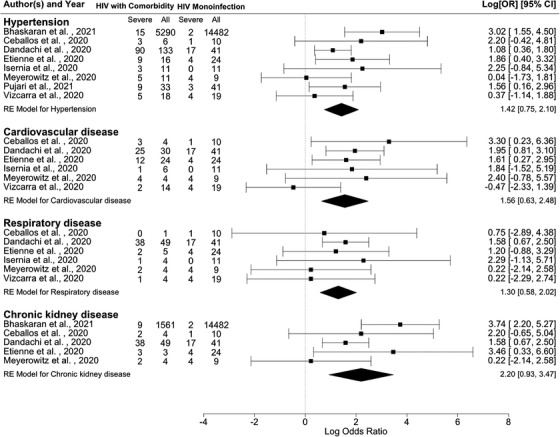
Forest plots of the associations of HIV/AANCC comorbidities in comparison to HIV monoinfection with severe COVID‐19 outcomes. Note. Hypertension: Heterogeneity: *Q* = 10.49, *df* = 7, *p* = 0.1627, *I* ^2^ = 36.41%; Cardiovascular disease: Heterogeneity: *Q* = 6.56, *df* = 5, *p* = 0.2551, *I* ^2^ = 26.79%; Respiratory disease: Heterogeneity: *Q* = 2.29, *df* = 5, *p* = 0.8073, *I* ^2^ = 0.00%; Chronic kidney disease: Heterogeneity: *Q* = 8.74, *df* = 4, *p* = 0.0679, *I* ^2^ = 55.01%. The scale on the x‐axis is the log odds ratio. Abbreviation: 95% CI, 95% confidence interval.

Figure 4 presents the pooled OR for selected AANCC comorbidities. Among the selected comorbidities, the pooled estimates show that PLHIV with AANCC comorbidities had a statistically significant higher risk of severe COVID‐19 outcomes (for HIV/HPT: OR = 4.14, 95% CI = 2.12–8.17, τ = 0.56, 95% CI 0–1.84, *I* ^2^ = 36.41%; for HIV/CVD: OR = 4.75, 95% CI 1.89–11.94, τ = 0.58, 95% CI 0–2.82, *I* ^2^ = 26.79%; for HIV/RD: OR = 3.67, 95% CI 1.79–7.54, τ = 0, 95% CI 0–1.43, *I* ^2^ = 0.00%; for HIV/CKD: OR = 9.02, 95% CI 2.53–32.14, τ = 1.02, 95% CI 0–3.96, *I* ^2^ = 55.01%).

**Figure 5:**
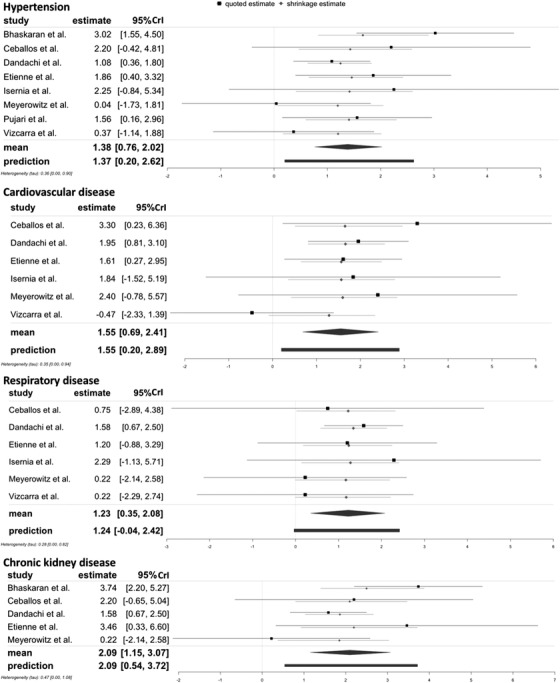
Forest plots of the association of HIV/AANCC comorbidities in comparison to HIV monoinfection with severe COVID‐19 outcomes with a Bayesian approach with half‐normal distribution prior (scale of 0.5). Note. The black line indicates the quoted estimates specified through effect from individual study and its σi as same as in the frequentist approach. The grey line presents the shrinkage intervals, which illustrate the posterior of each study's true effect. The black diamond presents the posterior distribution of the pooled effect, while the black bar shows the prediction distribution. The scale on the x‐axis is log odds ratio. Abbreviation: 95% CrI, 95% credible interval.

Figure 5 shows the forest plots estimated by the Bayesian approach for the other HIV/comorbidities combinations compared to HIV monoinfection. The Bayesian estimates for HIV/comorbidities were similar to the results from frequentist approach but with a more concise posterior distribution [Table 2, for HIV/HPT: OR = 3.97, 95% CrI = 2.14–7.54, τ = 0.36 (95% CrI 0–0.90); for HIV/CVD: OR = 4.71, 95% CrI = 1.99–11.13, τ = 0.35 (95% CrI 0–0.94); for HIV/RD: OR = 3.42, 95% CrI 1.42–8.00, τ = 0.28 (95% CrI 0–0.82); for HIV/CKD: OR = 8.08, 95% CrI 3.16–21.54, τ = 0.47 (95% CrI 0–1.08)]. Detailed information for the posterior distributions can be found in Appendix S7 through S10.

In terms of the predicted effect distributions for the future, the predicted effects for HIV/other comorbidities were similar to the posterior effects (Table 2, for HIV/HPT: OR = 3.94, 95% CrI = 1.22–13.74; for HIV/CVD: OR = 4.71, 95%CrI 1.22–17.99; for HIV/CKD: OR = 8.08, 95% CrI 1.72–41.26). Only the HIV/RD comorbidity showed a non‐significant probability of a higher risk to develop severe COVID‐19 outcomes compared to patients with an HIV monoinfection (OR = 3.46, 95% CrI 0.96–11.25).

There was no evidence of a publication bias for the risk of developing severe COVID‐19‐related outcomes among PLHIV with any selected AANCCs based on the funnel plots, mixed‐effects meta‐regression model and rank correction test. The publication bias assessment can be found in Appendix S4.

## 4 DISCUSSION

Not only is this first meta‐analysis that has a specific focus on PLHIV populations with and without comorbidities in the context of the COVID‐19 pandemic, but it also applies two different analytical methodologies of synthesis to explore the influence of between‐studies variance on the pooled results when the number of primary data available is limited. This comparative meta‐analysis of nine studies summarizes evidence that PLHIV with selected AANCCs had a higher risk, respectively, likelihood (depending on the meta‐analytic approach) to develop severe COVID‐19‐related outcomes compared to those who living with HIV monoinfection from both a classical frequentist approach and a Bayesian approach.

Our pooled likelihood findings corroborate previously obtained results on selected AANCCs as an at‐risk condition for severe COVID‐19‐related outcomes [1, 9, 10, 22]. PLHIV with diabetes had a higher likelihood of more than seven times (Table 2 and Figure 3) to develop severe outcomes after an infection with the COVID‐19. This estimated likelihood was in line with, yet almost two times higher than the pooled risk (OR = 3.68) reported by Zheng et al. When comparing people living with diabetes in the general population [1], it confirmed the argument of PLHIV with diabetes to be associated with a higher risk of severe COVID‐19 by Mellor et al. [9] with meta‐analytic evidence. Similarly, a higher likelihood (OR = 3.97) was observed in our study when comparing HIV/HTP with HIV monoinfection than the pooled risk (OR = 2.72) reported by Zheng et al. in the context without HIV infection [1]. This result was also in line with the evidence of a higher prevalence of HPT in severe cases of COVID‐19 among the general population without HIV by Gold et al. [2]. For HIV/CVD in comparison with an HIV monoinfection, we observed a higher likelihood of almost five times. This was similar to the pooled risk (OR = 5.19) reported by Zheng et al. [1]. However, these findings were contrary to the results from the meta‐regression conducted by Hariyanto et al., which indicated that CVD insignificantly affects the association between HIV and composite poor COVID‐19‐related outcomes compared to the general population [12]. In addition, it is noticeable that PLHIV with CKD have the highest likelihood of developing severe COVID‐19‐related outcomes compared to other AANCCs. The likelihood of HIV/RD in comparison with an HIV monoinfection (OR = 3.42) was lower than the pooled risks (OR = 5.15) reported by Zheng et al. [1]. For RDs, despite the resulting posterior medians and 95% CrI among PLHIV with AANCCs indicating a significant higher likelihood of developing a severe COVID‐19‐related outcome, the posterior distribution of the future observation predicted an insignificant likelihood of developing COVID‐19‐related outcomes. This result may thus disconfirm the expectation from Cooper et al. raised in their systematic review of more severe viral pneumonia outcomes among PLHIV with concurrent bacterial pneumonia [11]. This may be as a result of improved clinical management of RDs under the COVID‐19 pandemic, promoted by the World Health Organization in 2020 [36]. In sum, the prediction distributions among PLHIV with AANCCs stressed the need to focus on these sub‐populations living with HIV in a COVID‐19 clinical setting. Our meta‐analytic results show that PLHIV with AANCCs and coinfection with COVID‐19 need to receive extra attention to prevent and manage severe COVID‐19‐related outcomes.

To improve the stability of the effects by considering the influence from the random effects, we decided to include a Bayesian meta‐analytic approach and to compare the results with the classic frequentist meta‐analysis. One of the reasons of conducting this meta‐analysis with two methodologies was the small number of primary studies included at the given timepoint. Given the ongoing pandemic, postponing the meta‐analytic summary is not an option. Thus, in such a context, using the classical frequentist approach in meta‐analysis usually fails to pick‐up the random effect heterogeneity τ [37], which may lead to a biased result. In particular, our results confirmed this argument when the Bayesian approach succeeded to measure more concise random effects from the pooled studies compared to the classical frequentist approach (that failed to pick up the variance present in the data when, for example, PLHIV/RD are compared with HIV monoinfection). Moreover, generally, the heterogeneity τ was smaller in the Bayesian approach due to the assignment of the informative prior distribution.

There are marked differences in the range of intervals estimated between the classical frequentist approach and the Bayesian approach. Even though the pooled results estimated by these two different methodologies were similar, the CrIs generated by the Bayesian approach were generally much narrower than the CIs in the frequentist approach. This comparison allowed us to provide a more concise probability. Therefore, we recommend applying a Bayesian meta‐analytic approach in the context of limited primary data evidence. This way, more stable estimated fixed effects and random effects with a prediction distribution are available, and the posterior distribution can better inform any subsequent decision making [37].

Our review has several strengths and limitations. The major strength being the application of both the comparative frequentist and Bayesian meta‐analysis approaches. With the consistent estimated fixed effects, this review presents a solid argument for the importance of extra care for PLHIV with AANCCs. Also, the introduction of Bayesian meta‐analysis in our study proved the feasibility and durability of this methodology in the field of HIV, owing to the fact that studies related to HIV are often heterogeneous. Another strength of this study is that we reported new pooled estimates sorely focusing on the PLHIV population. The results from this review could substantially inform the clinical management of PLHIV who are coinfected with COVID‐19.

One of the limitations was the broad definition of severe COVID‐19 outcomes. Due to the lack of global standardized classifications of severe COVID‐19‐related outcomes, data from studies which reported severe COVID‐19‐related outcomes were thus heterogeneous. This heterogeneity of the definition of severe COVID‐19‐related outcomes may thus have had an impact on data grouping in this review and lead to an increase in bias. However, the application of Bayesian meta‐analysis compensates this limitation as much as possible. Another limitation of this meta‐analysis may be the lack of sub‐group analysis in terms of gender, or other minor population characteristics. All except one of the included studies in this meta‐analysis did not provide information on the gender proportion when reporting on PLHIV with AANCCs [7, 13, 29, 30, 31, 32, 33]. Our studies thus cannot exclude the possibility of an influence of gender differences on the posterior distribution of the association between PLHIV with AANCCs and HIV monoinfection, given the fact that women were found less susceptible to viral‐infection than men due to the differences in innate and adaptive immunity protection from the X chromosome and sex hormones [38]. Therefore, we stress a need of including gender information when reporting on PLHIV with AANCCs in the COVID‐19 context. Studies which reported HIV/COVID‐19 coinfection among minors were not included in this meta‐analysis due to a lack of data. We thus cannot draw any conclusion for the likelihood of the severe COVID‐19‐related outcomes among young PLHIV with various comorbidities. Given the non‐ignorable size of adolescents living with HIV and under the risk of a COVID‐19 co‐infection, future meta‐analytic research is warranted when the primary data become available. In addition, in our meta‐analysis, the missing information on HIV viral load in the studies included in this meta‐analysis may be seem as another limitation. Higher HIV viral load was suggested to be a risk factor for severe COVID‐19 in PLHIV in some [39] but not all studies [7, 9]. Results from this meta‐analysis may be biased when applied to PLHIV population with different viral loads. Hence, we recommend future studies to include the information on HIV virus load when reporting their parameters. One more limitation in this meta‐analysis may be that we did not perform an analysis on PLHIV with multiple AANCCs, again due to the lack of primary data. This may limit the scope of the posterior distribution estimated in this study and resulting in underestimation of the posterior median for PLHIV with multiple different AANCCs. In sum, more detailed primary data reporting has the potential to improve the quality of future research syntheses and would allow to draw even more robust conclusions.

## 5 CONCLUSIONS

In conclusion, there is an increased likelihood of developing severe COVID‐19‐related outcomes among PLHIV who are experiencing a COVID‐19 coinfection, and who are living with an AANCC, such as diabetes, HPT, CVD, RD or CKD. The application of a Bayesian meta‐analysis improved the stability of the estimated effects with a more concise random effect estimation to compensate the heterogeneity of the included studies. Healthcare providers need to be aware of the severe COVID‐19 progression risk among their clients living with HIV and an AANCC. Public health policy makers should adjust vaccination priorities based on these findings. Especially in countries in which certain parts of the population are less willing to get vaccinated, such as some states within the United States of America [40, 41, 42], healthcare providers working with PLHIV should inform their clients based on their AANCC comorbidities about the advantages of a vaccination.

## COMPETING INTERESTS

The authors declare no competing interests.

## AUTHORS’ CONTRIBUTION

HW and KJJ conceptualized this review and planned the analysis. HW independently did the screening and extracted data and all the analysis. KJJ double‐checked data extraction and checked the data analysis. HW and KJJ interpreted the results and conceptualized the first draft of the review. All authors read and approved the final version of the manuscript.

## FUNDING

There was no funding source for this study.

## Supporting information


**Table S1**. PRISMA checklist
**Table S2**. Search terms for different databases
**Table S3**. Study characteristics
**Figure S4**. Publication bias (Funnel‐plot analysis) for the association of HIV/comorbidity in comparison to HIV monoinfection with severe COVID‐19 outcomes
**Figure S5**. Sensitivity analysis of different scales of half‐normal distribution using HIV/Diabetes sub‐group analysis
**Figure S6**. Posterior distribution of the fixed effects and random effects τ for the association of HIV/Diabetes in comparison to HIV monoinfection with severe COVID‐19 outcomes with a Bayesian approach with half normal distribution prior (scale of 0.5)
**Figure S7**. Posterior distribution of the fixed effects and random effects τ for the association of HIV/Hypertension in comparison to HIV monoinfection with severe COVID‐19 outcomes with a Bayesian approach with half normal distribution prior (scale of 0.5)
**Figure S8**. Posterior distribution of the fixed effects and random effects τ for the association of HIV/Cardiovascular disease in comparison to HIV monoinfection with severe COVID‐19 outcomes with a Bayesian approach with half normal distribution prior (scale of 0.5)
**Figure S9**. Posterior distribution of the fixed effects and random effects τ for the association of HIV/Respiratory disease in comparison to HIV monoinfection with severe COVID‐19 outcomes with a Bayesian approach with half normal distribution prior (scale of 0.5)
**Figure S10**. Posterior distribution of the fixed effects and random effects τ for the association of HIV/Chronic kidney disease in comparison to HIV monoinfection with severe COVID‐19 outcomes with a Bayesian approach with half normal distribution prior (scale of 0.5)

## Data Availability

Data is available on request from the authors.
