# Supplementary material for: The likelihood of severe COVID‐19 outcomes among PLHIV with various comorbidities: a comparative frequentist and Bayesian meta‐analysis approach
Source: J Int AIDS Soc. 2021 Nov 19;24(11):e25841. doi: 10.1002/jia2.25841 (PMC8604378; doi:10.1002/jia2.25841)
Supplement: Supplementary file 1 — Table S1. PRISMA checklist Table S2. Search terms for different databases Table S3. Study characteristics Figure S4. Publication bias (Funnel‐plot analysis) for the association of HIV/comorbidity in comparison to HIV monoinfection with severe COVID‐19 outcomes Figure S5. Sensitivity analysis of different scales of half‐normal distribution using HIV/Diabetes sub‐group analysis Figure S6. Posterior distribution of the fixed effects and random effects τ for the association of HIV/Diabetes in comparison to HIV monoinfection with severe COVID‐19 outcomes with a Bayesian approach with half normal distribution prior (scale of 0.5) Figure S7. Posterior distribution of the fixed effects and random effects τ for the association of HIV/Hypertension in comparison to HIV monoinfection with severe COVID‐19 outcomes with a Bayesian approach with half normal distribution prior (scale of 0.5) Figure S8. Posterior distribution of the fixed effects and random effects τ for the association of HIV/Cardiovascular disease in comparison to HIV monoinfection with severe COVID‐19 outcomes with a Bayesian approach with half normal distribution prior (scale of 0.5) Figure S9. Posterior distribution of the fixed effects and random effects τ for the association of HIV/Respiratory disease in comparison to HIV monoinfection with severe COVID‐19 outcomes with a Bayesian approach with half normal distribution prior (scale of 0.5) Figure S10. Posterior distribution of the fixed effects and random effects τ for the association of HIV/Chronic kidney disease in comparison to HIV monoinfection with severe COVID‐19 outcomes with a Bayesian approach with half normal distribution prior (scale of 0.5) [file JIA2-24-e25841-s001.docx]

# Appendix

**Table S1 PRISMA checklist**

| **Section/topic** | **#** | **Checklist item** | **Reported on page #** |
| --- | --- | --- | --- |
| **TITLE** | | |  |
| Title | 1 | Identify the report as a systematic review, meta-analysis, or both. | 1 |
| **ABSTRACT** | | |  |
| Structured summary | 2 | Provide a structured summary including, as applicable: background; objectives; data sources; study eligibility criteria, participants, and interventions; study appraisal and synthesis methods; results; limitations; conclusions and implications of key findings; systematic review registration number. | 2 |
| **INTRODUCTION** | | |  |
| Rationale | 3 | Describe the rationale for the review in the context of what is already known. | 3 |
| Objectives | 4 | Provide an explicit statement of questions being addressed with reference to participants, interventions, comparisons, outcomes, and study design (PICOS). | 3 |
| **METHODS** | | |  |
| Protocol and registration | 5 | Indicate if a review protocol exists, if and where it can be accessed (e.g., Web address), and, if available, provide registration information including registration number. | NA |
| Eligibility criteria | 6 | Specify study characteristics (e.g., PICOS, length of follow-up) and report characteristics (e.g., years considered, language, publication status) used as criteria for eligibility, giving rationale. | 4 |
| Information sources | 7 | Describe all information sources (e.g., databases with dates of coverage, contact with study authors to identify additional studies) in the search and date last searched. | 3 |
| Search | 8 | Present full electronic search strategy for at least one database, including any limits used, such that it could be repeated. | 3 |
| Study selection | 9 | State the process for selecting studies (i.e., screening, eligibility, included in systematic review, and, if applicable, included in the meta-analysis). | 4 |
| Data collection process | 10 | Describe method of data extraction from reports (e.g., piloted forms, independently, in duplicate) and any processes for obtaining and confirming data from investigators. | 4 |
| Data items | 11 | List and define all variables for which data were sought (e.g., PICOS, funding sources) and any assumptions and simplifications made. | 4 |
| Risk of bias in individual studies | 12 | Describe methods used for assessing risk of bias of individual studies (including specification of whether this was done at the study or outcome level), and how this information is to be used in any data synthesis. | 4 |
| Summary measures | 13 | State the principal summary measures (e.g., risk ratio, difference in means). | 4 |
| Synthesis of results | 14 | Describe the methods of handling data and combining results of studies, if done, including measures of consistency (e.g., I^2^) for each meta-analysis. | 4 |

| Risk of bias across studies | 15 | Specify any assessment of risk of bias that may affect the cumulative evidence (e.g., publication bias, selective reporting within studies). | 4 |
| --- | --- | --- | --- |
| Additional analyses | 16 | Describe methods of additional analyses (e.g., sensitivity or subgroup analyses, meta-regression), if done, indicating which were pre-specified. | 4 |
| **RESULTS** | | |  |
| Study selection | 17 | Give numbers of studies screened, assessed for eligibility, and included in the review, with reasons for exclusions at each stage, ideally with a flow diagram. | 4 |
| Study characteristics | 18 | For each study, present characteristics for which data were extracted (e.g., study size, PICOS, follow-up period) and provide the citations. | 8,  Appendix 3 |
| Risk of bias within studies | 19 | Present data on risk of bias of each study and, if available, any outcome level assessment (see item 12). | 5 |
| Results of individual studies | 20 | For all outcomes considered (benefits or harms), present, for each study: (a) simple summary data for each intervention group (b) effect estimates and confidence intervals, ideally with a forest plot. | 8 |
| Synthesis of results | 21 | Present results of each meta-analysis done, including confidence intervals and measures of consistency. | 8 |
| Risk of bias across studies | 22 | Present results of any assessment of risk of bias across studies (see Item 15). | Appendix 7 |
| Additional analysis | 23 | Give results of additional analyses, if done (e.g., sensitivity or subgroup analyses, meta-regression [see Item 16]). | 7 |
| **DISCUSSION** | | |  |
| Summary of evidence | 24 | Summarize the main findings including the strength of evidence for each main outcome; consider their relevance to key groups (e.g., healthcare providers, users, and policy makers). | 13 |
| Limitations | 25 | Discuss limitations at study and outcome level (e.g., risk of bias), and at review-level (e.g., incomplete retrieval of identified research, reporting bias). | 14 |
| Conclusions | 26 | Provide a general interpretation of the results in the context of other evidence, and implications for future research. | 13 |
| **FUNDING** | | |  |
| Funding | 27 | Describe sources of funding for the systematic review and other support (e.g., supply of data); role of funders for the systematic review. | 16 |

**Table S2. Search terms for different databases**

| **Keywords** | | |
| --- | --- | --- |
| **HIV** | **Comorbidities** | **COVID-19 outcomes** |
| “Human immunodeficiency virus” OR HIV | Comorbidity OR Diabetes OR Hypertension OR “Cardiovascular disease” “Respiratory disease”  OR “Pulmonary disease” OR “Chronic kidney disease” OR “Impaired renal function” | (“COVID-19” OR “SARS-CoV-2” OR Coronavirus) AND (Hospitalisation OR  “Severe symptoms” OR “Intensive care” OR Incubation OR “Assisted ventilation” OR Death OR Deceased) |
| **PubMed search strategy** First search conducted 23-02-2021 1,687 articles retrieved  Second search conducted 25-06-2021 403 articles retrieved, timespan=24-02-2021 to 25-06-2021 | | |
| **1. HIV/Comorbidity:** (("hiv"[MeSH Terms] OR "hiv"[All Fields] OR “Human immunodeficiency virus”[All Field]) AND ("comorbid"[All Fields] OR "comorbidity"[MeSH Terms] OR "comorbidity"[All Fields] OR "comorbidities"[All Fields] OR "comorbids"[All Fields]) AND ("covid 19"[All Fields] OR "covid 19"[MeSH Terms] OR "covid 19 vaccines"[All Fields] OR "covid 19 vaccines"[MeSH Terms] OR "covid 19 serotherapy"[All Fields] OR "covid 19 serotherapy"[Supplementary Concept] OR "covid 19 nucleic acid testing"[All Fields] OR "covid 19 nucleic acid testing"[MeSH Terms] OR "covid 19 serological testing"[All Fields] OR "covid 19 serological testing"[MeSH Terms] OR "covid 19 testing"[All Fields] OR "covid 19 testing"[MeSH Terms] OR "sars cov 2"[All Fields] OR "sars cov 2"[MeSH Terms] OR "severe acute respiratory syndrome coronavirus 2"[All Fields] OR "ncov"[All Fields] OR "2019 ncov"[All Fields] OR (("coronavirus"[MeSH Terms] OR "coronavirus"[All Fields] OR "cov"[All Fields])) | | |
| **2. HIV/Diabetes**: (("hiv"[MeSH Terms] OR "hiv"[All Fields]) AND ("diabete"[All Fields] OR "diabetes mellitus"[MeSH Terms] OR ("diabetes"[All Fields] AND "mellitus"[All Fields]) OR "diabetes mellitus"[All Fields] OR "diabetes"[All Fields] OR "diabetes insipidus"[MeSH Terms] OR ("diabetes"[All Fields] AND "insipidus"[All Fields]) OR "diabetes insipidus"[All Fields] OR "diabetic"[All Fields] OR "diabetics"[All Fields] OR "diabets"[All Fields]) AND ("covid 19"[All Fields] OR "covid 19"[MeSH Terms] OR "covid 19 vaccines"[All Fields] OR "covid 19 vaccines"[MeSH Terms] OR "covid 19 serotherapy"[All Fields] OR "covid 19 serotherapy"[Supplementary Concept] OR "covid 19 nucleic acid testing"[All Fields] OR "covid 19 nucleic acid testing"[MeSH Terms] OR "covid 19 serological testing"[All Fields] OR "covid 19 serological testing"[MeSH Terms] OR "covid 19 testing"[All Fields] OR "covid 19 testing"[MeSH Terms] OR "sars cov 2"[All Fields] OR "sars cov 2"[MeSH Terms] OR "severe acute respiratory syndrome coronavirus 2"[All Fields] OR "ncov"[All Fields] OR "2019 ncov"[All Fields] OR (("coronavirus"[MeSH Terms] OR "coronavirus"[All Fields] OR "cov"[All Fields])) | | |
| **3. HIV/Hypertension**: (("hiv"[MeSH Terms] OR "hiv"[All Fields]) AND ("hypertense"[All Fields] OR "hypertension"[MeSH Terms] OR "hypertension"[All Fields] OR "hypertension s"[All Fields] OR "hypertensions"[All Fields] OR "hypertensive"[All Fields] OR "hypertensive s"[All Fields] OR  "hypertensives"[All Fields]) AND ("covid 19"[All Fields] OR "covid 19"[MeSH Terms] OR "covid 19 vaccines"[All Fields] OR "covid 19 vaccines"[MeSH Terms] OR "covid 19 serotherapy"[All Fields] OR "covid 19 serotherapy"[Supplementary Concept] OR "covid 19 nucleic acid testing"[All Fields] OR "covid 19 nucleic acid testing"[MeSH Terms] OR "covid 19 serological testing"[All Fields] OR "covid 19 serological testing"[MeSH Terms] OR "covid 19 testing"[All Fields] OR "covid 19 testing"[MeSH Terms] OR "sars cov 2"[All Fields] OR "sars cov 2"[MeSH Terms] OR "severe acute respiratory syndrome coronavirus 2"[All Fields] OR "ncov"[All Fields] OR "2019 ncov"[All Fields] OR (("coronavirus"[MeSH Terms] OR "coronavirus"[All Fields] OR "cov"[All Fields])) | | |
| **4. HIV/Cardiovascular disease:** (("hiv"[MeSH Terms] OR "hiv"[All Fields]) AND ("cardiovascular diseases"[MeSH Terms] OR ("cardiovascular"[All Fields] AND "diseases"[All Fields]) OR "cardiovascular diseases"[All Fields] OR ("cardiovascular"[All Fields] AND "disease"[All Fields]) OR "cardiovascular  disease"[All Fields]) AND ("covid 19"[All Fields] OR "covid 19"[MeSH Terms] OR "covid 19 vaccines"[All Fields] OR "covid 19 vaccines"[MeSH Terms] OR "covid 19 serotherapy"[All Fields] OR "covid 19 serotherapy"[Supplementary Concept] OR "covid 19 nucleic acid testing"[All Fields] OR "covid 19 nucleic acid testing"[MeSH Terms] OR "covid 19 serological testing"[All Fields] OR "covid 19 serological testing"[MeSH Terms] OR "covid 19 testing"[All Fields] OR "covid 19 testing"[MeSH Terms] OR "sars cov 2"[All Fields] OR "sars cov 2"[MeSH Terms] OR "severe acute respiratory syndrome coronavirus 2"[All Fields] OR "ncov"[All Fields] OR "2019 ncov"[All Fields] OR (("coronavirus"[MeSH Terms] OR "coronavirus"[All Fields] OR "cov"[All Fields])) | | |
| **5. HIV/Respiratory disease:** (("hiv"[MeSH Terms] OR "hiv"[All Fields]) AND ("respiratory tract diseases"[MeSH Terms] OR ("respiratory"[All Fields] AND "tract"[All Fields] AND "diseases"[All Fields]) OR "respiratory tract diseases"[All Fields] OR ("respiratory"[All Fields] AND "disease"[All Fields]) OR "respiratory disease"[All Fields] OR "respiration disorders"[MeSH Terms] OR ("respiration"[All Fields] AND "disorders"[All Fields]) OR "respiration disorders"[All Fields] OR ("respiratory"[All Fields] AND "disease"[All Fields])) AND ("covid 19"[All Fields] OR "covid 19"[MeSH Terms] OR "covid 19 vaccines"[All Fields] OR "covid 19 vaccines"[MeSH Terms] OR "covid 19 serotherapy"[All Fields] OR "covid 19 serotherapy"[Supplementary Concept] OR "covid 19 nucleic acid testing"[All Fields] OR "covid 19 nucleic acid testing"[MeSH Terms] OR "covid 19 serological testing"[All Fields] OR "covid 19 serological testing"[MeSH Terms] OR "covid 19 testing"[All Fields] OR "covid 19 testing"[MeSH Terms] OR "sars cov 2"[All Fields] OR "sars cov 2"[MeSH Terms] OR "severe acute respiratory syndrome coronavirus 2"[All Fields] OR "ncov"[All Fields] OR "2019 ncov"[All Fields] OR (("coronavirus"[MeSH Terms] OR "coronavirus"[All Fields] OR "cov"[All Fields])) **AND**: 5. (("hiv"[MeSH Terms] OR "hiv"[All Fields]) AND ("lung diseases"[MeSH Terms] OR ("lung"[All Fields] AND "diseases"[All Fields]) OR "lung diseases"[All Fields] OR ("pulmonary"[All Fields] AND "disease"[All Fields]) OR "pulmonary disease"[All Fields]) AND ("covid 19"[All Fields] OR "covid 19"[MeSH Terms] OR "covid 19 vaccines"[All Fields] OR "covid 19 vaccines"[MeSH Terms] OR "covid 19 serotherapy"[All Fields] OR "covid 19 serotherapy"[Supplementary Concept] OR "covid 19 nucleic acid testing"[All Fields] OR "covid 19 nucleic acid testing"[MeSH Terms] OR "covid 19 serological testing"[All Fields] OR "covid 19 serological testing"[MeSH Terms] OR "covid 19 testing"[All Fields] OR "covid 19 testing"[MeSH Terms] OR "sars cov 2"[All Fields] OR "sars cov 2"[MeSH Terms] OR "severe acute respiratory syndrome coronavirus 2"[All Fields] OR "ncov"[All Fields] OR "2019 ncov"[All Fields] OR (("coronavirus"[MeSH Terms] OR "coronavirus"[All Fields] OR "cov"[All Fields])) | | |
| **6. HIV/Chronic kidney disease:** (("hiv"[MeSH Terms] OR "hiv"[All Fields]) AND ("renal insufficiency, chronic"[MeSH Terms] OR ("renal"[All Fields] AND "insufficiency"[All Fields] AND "chronic"[All Fields]) OR "chronic renal insufficiency"[All Fields] OR ("chronic"[All Fields] AND "kidney"[All Fields] AND "disease"[All Fields]) OR "chronic kidney disease"[All Fields]) AND ("covid 19"[All Fields] OR "covid 19"[MeSH Terms] OR "covid 19 vaccines"[All Fields] OR "covid 19 vaccines"[MeSH Terms] OR "covid 19 serotherapy"[All Fields] OR "covid 19 serotherapy"[Supplementary Concept] OR "covid 19 nucleic acid testing"[All Fields] OR "covid 19 nucleic acid testing"[MeSH Terms] OR "covid 19 serological testing"[All Fields] OR "covid 19 serological testing"[MeSH Terms] OR "covid 19 testing"[All Fields] OR "covid 19 testing"[MeSH Terms] OR "sars cov 2"[All Fields] OR "sars cov 2"[MeSH Terms] OR "severe acute respiratory syndrome coronavirus 2"[All Fields] OR "ncov"[All Fields] OR "2019 ncov"[All Fields] OR (("coronavirus"[MeSH Terms] OR "coronavirus"[All Fields] OR "cov"[All Fields]) AND 2019/11/01:3000/12/31[Date - Publication]))) AND ((fha[Filter]) AND (fft[Filter]) **AND:** (("hiv"[MeSH Terms] OR "hiv"[All Fields]) AND ("renal insufficiency"[MeSH Terms] OR ("renal"[All Fields] AND "insufficiency"[All Fields]) OR "renal insufficiency"[All Fields] OR ("impaired"[All Fields] AND "renal"[All Fields] AND "function"[All Fields]) OR "impaired renal function"[All Fields]) AND ("covid 19"[All Fields] OR "covid 19"[MeSH Terms] OR "covid 19 vaccines"[All Fields] OR "covid 19 vaccines"[MeSH Terms] OR "covid 19 serotherapy"[All Fields] OR "covid 19 serotherapy"[Supplementary Concept] OR "covid 19 nucleic acid testing"[All Fields] OR "covid 19 nucleic acid testing"[MeSH Terms] OR "covid 19 serological testing"[All Fields] OR "covid 19 serological testing"[MeSH Terms] OR "covid 19 testing"[All Fields] OR "covid 19 testing"[MeSH Terms] OR "sars cov 2"[All Fields] OR "sars cov 2"[MeSH Terms] OR "severe acute respiratory syndrome coronavirus 2"[All Fields] OR "ncov"[All Fields] OR "2019 ncov"[All Fields] OR (("coronavirus"[MeSH Terms] OR "coronavirus"[All Fields] OR "cov"[All Fields])) | | |
| **Web of Science search strategy** First search conducted 23-02-2021 373 articles retrieved  Second search conducted 25-06-2021 108 articles retrieved | | |
| (TS=HIV* OR TS="Human immunodeficiency virus") AND (TS=comorbidity* OR TS=diabetes* OR TS=hypertension* OR TS="cardiovascular disease" OR TS="respiratory disease" OR TS="pulmonary disease" OR TS="chronic kidney disease" OR TS="impaired renal dysfunction") AND (TS="COVID-19" OR TS="SARS-CoV-2" OR TS=hospitalisation* OR TS="severe symptoms" OR TS="Intensive care" OR TS="assisted ventilation" OR TS=death* OR TS=mortality*) | | |
| AND Timespan=2020-2021  AND Timespan= 2021 (for the second search) | | |
| medRxiv search strategy First search conducted 25-06-2021 2 articles retrieved | | |
| (HIV* OR "Human immunodeficiency virus") AND (comorbidity* OR diabetes* OR hypertension* OR "cardiovascular disease" OR "respiratory disease" OR "pulmonary disease" OR "chronic kidney disease" OR "impaired renal dysfunction") AND ("COVID-19" OR "SARS-CoV-2" OR hospitalisation* OR "severe symptoms" OR "Intensive care" OR "assisted ventilation" OR death* OR mortality*) | | |
| AND Timespan= 24-02-2021 to 25-06-2021 | | |

**Table S3. Study characteristics**

| **Comorbidity** | **Study** | **Year** | **Country** | **Sample size** | **COVID-19 outcome** | **Number of severe outcome among HIV and comorbidity** | **Number of HIV and comorbidity** | **Number of severe outcome  among HIV monoinfection** | **Number of HIV monoinfection** |
| --- | --- | --- | --- | --- | --- | --- | --- | --- | --- |
| **Diabetes** | Bhaskaran et al. ^1^ | 2021 | UK | 27480 | Mortality | 14 (0.51%) | 2709 | 2 (0.01%) | 14482 |
|  | Boulle et al. ^2^ | 2020 | South Africa | 3978 | Mortality | 58 (13.49%) | 430 | 57 (1.61%) | 3548 |
|  | Ceballos et al. ^3^ | 2020 | Chile | 36 | Mortality | 2 (50.00%) | 4 | 1 (10.00%) | 10 |
|  | Dandachi et al. ^4^ | 2020 | US | 286 | Hospitalisation | 42 (68.85%) | 61 | 17 (41.46%) | 41 |
|  | Etienne et al. ^5^ | 2020 | France | 54 | Severe and critical | 5 (10 0.00%) | 5 | 4 (16.67%) | 24 |
|  | Isernia et al. ^6^ | 2020 | France | 30 | Hospitalisation | 4 (44.44%) | 9 | 0 (0.00%) | 11 |
|  | Meyerowitz et al. ^7^ | 2020 | US | 36 | Hospitalisation | 5 (62.50%) | 8 | 4 (44.44%) | 9 |
|  | Pujari et al. ^8^ | 2021 | India | 86 | Severe and critical | 4 (26.67%) | 15 | 3 (7.32%) | 41 |
|  | Vizcarra et al. ^9^ | 2020 | Spain | 51 | Severe and critical | 1 (14.29%) | 7 | 4 (21.05%) | 19 |
| **Hypertension** | Bhaskaran et al. ^1^ | 2021 | UK | 27480 | Mortality | 15 (0.28%) | 5290 | 2 (0.01%) | 14482 |
|  | Ceballos et al. ^3^ | 2020 | Chile | 36 | Mortality | 3 (50.00%) | 6 | 1 (10.00%) | 10 |
|  | Dandachi et al. ^4^ | 2020 | US | 286 | Hospitalisation | 90 (67.67%) | 133 | 17 (41.46%) | 41 |
|  | Etienne et al. ^5^ | 2020 | France | 54 | Severe and critical | 9 (56.25%) | 16 | 4 (16.67%) | 24 |
|  | Isernia et al. ^6^ | 2020 | France | 30 | Hospitalisation | 3 (27.27%) | 11 | 0 (0.00%) | 11 |
|  | Meyerowitz et al. ^7^ | 2020 | US | 36 | Hospitalisation | 5 (45.45%) | 11 | 4 (44.44%) | 9 |
|  | Pujari et al. ^8^ | 2021 | India | 86 | Severe and critical | 9 (27.27%) | 33 | 3 (7.32%) | 41 |
|  | Vizcarra et al. ^9^ | 2020 | Spain | 51 | Severe and critical | 5 (27.78%) | 18 | 4 (21.05%) | 19 |
| **Cardiovascular Disease** | Ceballos et al. ^3^ | 2020 | Chile | 36 | Mortality | 3 (75.00%) | 4 | 1 (10.00%) | 10 |
|  | Dandachi et al. ^4^ | 2020 | US | 286 | Hospitalisation | 25 (83.33%) | 30 | 17 (41.46%) | 41 |
|  | Etienne et al. ^5^ | 2020 | France | 54 | Severe and critical | 12 (50.00%) | 24 | 4 (16.67%) | 24 |
|  | Isernia et al. ^6^ | 2020 | France | 30 | Hospitalisation | 1 (16.67%) | 6 | 0 (0.00%) | 11 |
|  | Meyerowitz et al. ^7^ | 2020 | US | 36 | Hospitalisation | 4 (100.00%) | 4 | 4 (44.44%) | 9 |
|  | Vizcarra et al. ^9^ | 2020 | Spain | 51 | Severe and critical | 2 (14.29%) | 14 | 4 (21.05%) | 19 |
| **Respiratory Disease** | Ceballos et al. ^3^ | 2020 | Chile | 36 | Mortality | 0 (0.00%) | 1 | 1 (10.00%) | 10 |
|  | Dandachi et al. ^4^ | 2020 | US | 286 | Hospitalisation | 38 (77.55%) | 49 | 17 (41.46%) | 41 |
|  | Etienne et al. ^5^ | 2020 | France | 54 | Severe and critical | 2 (40.00%) | 5 | 4 (16.67%) | 24 |
|  | Isernia et al. ^6^ | 2020 | France | 30 | Hospitalisation | 1 (25.00%) | 4 | 0 (0.00%) | 11 |
|  | Meyerowitz et al. ^7^ | 2020 | US | 36 | Hospitalisation | 2 (50.00%) | 4 | 4 (44.44%) | 9 |
|  | Vizcarra et al. ^9^ | 2020 | Spain | 51 | Severe and critical | 1 (25.00%) | 4 | 4 (21.05%) | 19 |
| **Chronic kidney Disease** | Bhaskaran et al. ^1^ | 2021 | UK | 27480 | Death | 9 (0.58%) | 1561 | 2 (0.01%) | 14482 |
|  | Ceballos et al. ^3^ | 2020 | Chile | 36 | Mortality | 2 (50.00%) | 4 | 1 (10.00%) | 10 |
|  | Dandachi et al. ^4^ | 2020 | US | 286 | Hospitalisation | 38 (77.55%) | 49 | 17 (41.46%) | 41 |
|  | Etienne et al. ^5^ | 2020 | France | 54 | Severe and critical | 3 (100.00%) | 3 | 4 (16.67%) | 24 |
|  | Meyerowitz et al. ^7^ | 2020 | US | 36 | Hospitalisation | 2 (50.00%) | 4 | 4 (44.44%) | 9 |

**Figure S4 Publication bias (Funnel-plot analysis) for the association of HIV/comorbidity in comparison to HIV monoinfection with severe COVID-19 outcomes**

The funnel-plot analysis in this study indicating no publication bias for the five HIV/comorbidities compared to HIV monoinfection.


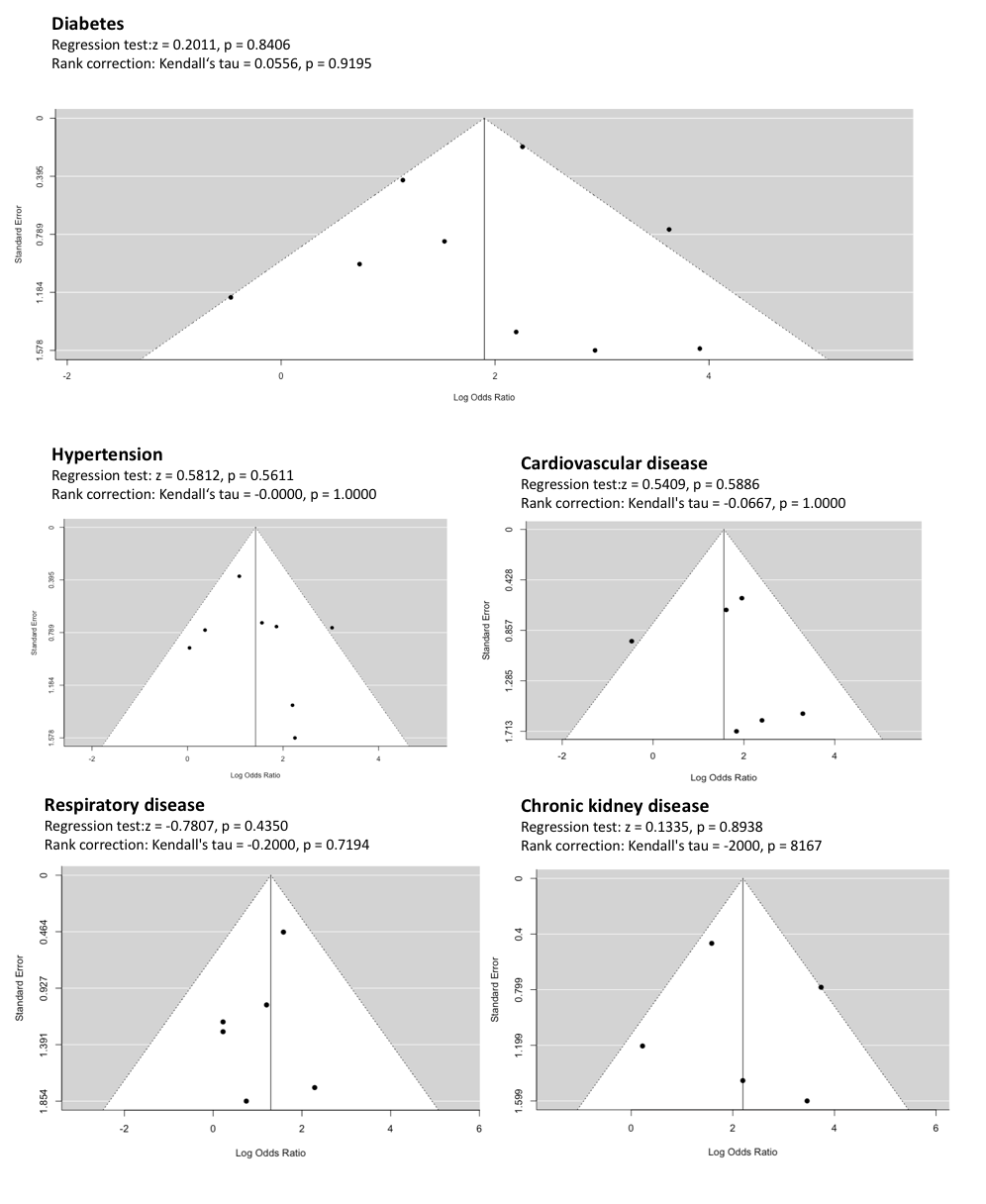


**Figure S5. Sensitivity analysis of different scales of half-normal distribution using HIV/Diabetes sub-group analysis**

We conducted a sensitivity analysis of different scales of the prior distribution using data from HIV plus diabetes population in comparison to HIV monoinfection. Results from the sensitivity analysis show that compared to half-normal distribution prior with scale of 0.5, results from half-normal distribution prior with scale 1.0 and half-Cauchy distribution prior with scale of 0.5 were more extreme. For consistent results in this meta-analysis, all other models with Bayesian approach used half-normal distribution prior with scale of 0.5.

**
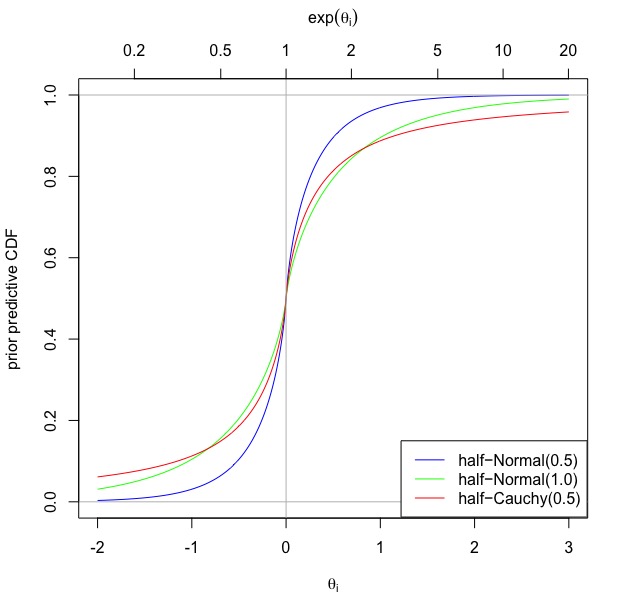
**

**Figure S6 Posterior distribution of the fixed effects and random effects τ for the association of HIV/Diabetes in comparison to HIV monoinfection with severe COVID-19 outcomes with a Bayesian approach with half normal distribution prior (scale of 0.5).**

(A) is a simple forest plot showing estimates and 95% intervals illustrating the input data along with the estimated mean eﬀect µ and a prediction interval for the eﬀect θ_k+1_ in a future study. (B) illustrates the joint posterior density of heterogeneity τ and eﬀect µ, with darker shading corresponding to higher probability density. The red lines indicate (approximate) 2-dimensional credible regions, and the green lines show marginal posterior medians and 95% credible intervals. The blue lines show the conditional posterior mean eﬀect µ(τ) as a function of the heterogeneity τ along with a 95% interval based on its conditional standard error σ (τ). The red cross (+) indicates the posterior mode, while the pink cross (×) shows the ML estimate. The two bottom plots (C) and (D) show the marginal posterior densities of eﬀect µ and heterogeneity τ. 95% credible intervals are indicated with a darker shading, and the posterior median is shown by a vertical line.


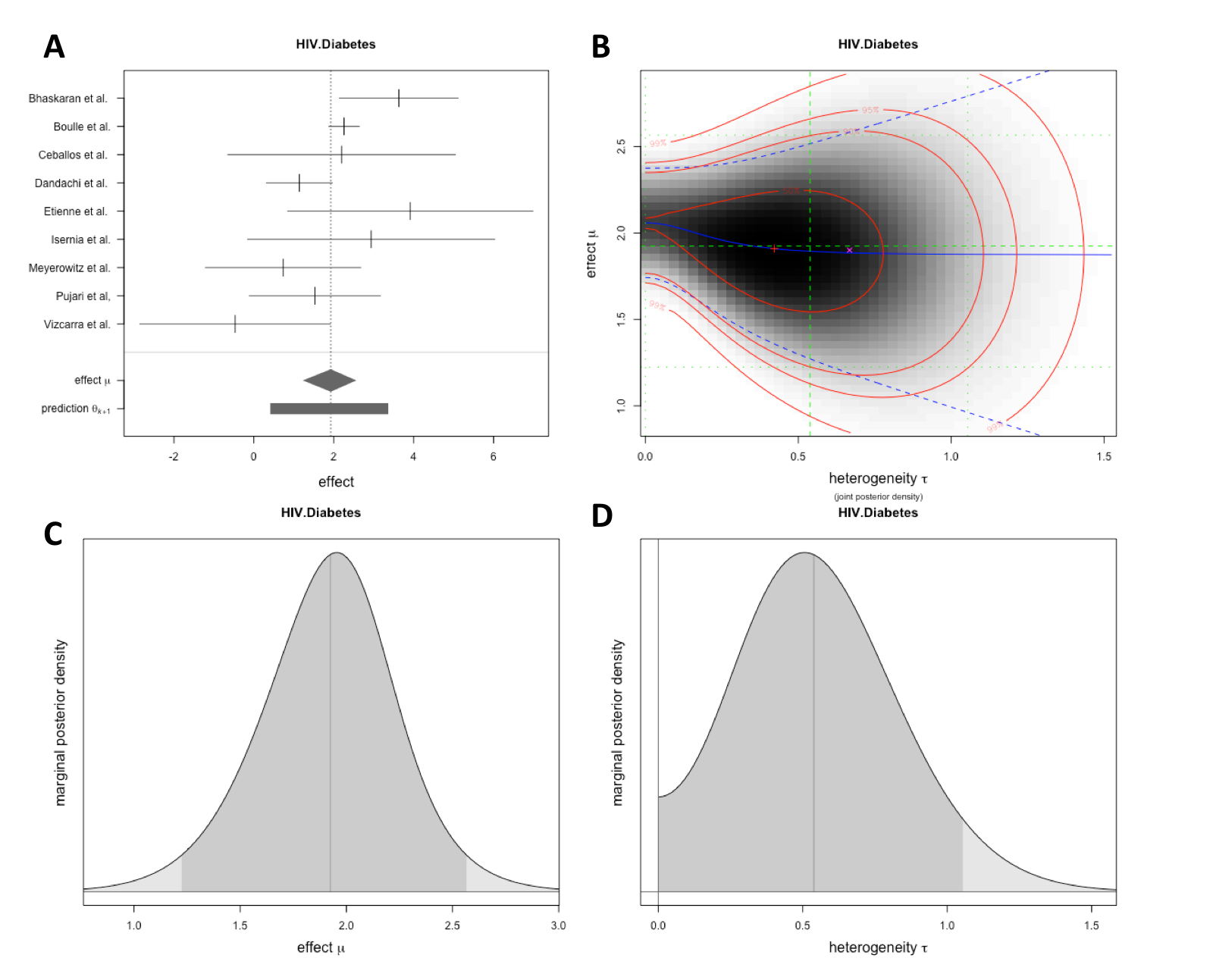


**Figure S7 Posterior distribution of the fixed effects and random effects τ for the association of HIV/Hypertension in comparison to HIV monoinfection with severe COVID-19 outcomes with a Bayesian approach with half normal distribution prior (scale of 0.5).**

(A) is a simple forest plot showing estimates and 95% intervals illustrating the input data along with the estimated mean eﬀect µ and a prediction interval for the eﬀect θ_k+1_ in a future study. (B) illustrates the joint posterior density of heterogeneity τ and eﬀect µ, with darker shading corresponding to higher probability density. The red lines indicate (approximate) 2-dimensional credible regions, and the green lines show marginal posterior medians and 95% credible intervals. The blue lines show the conditional posterior mean eﬀect µ(τ) as a function of the heterogeneity τ along with a 95% interval based on its conditional standard error σ (τ). The red cross (+) indicates the posterior mode, while the pink cross (×) shows the ML estimate. The two bottom plots (C) and (D) show the marginal posterior densities of eﬀect µ and heterogeneity τ. 95% credible intervals are indicated with a darker shading, and the posterior median is shown by a vertical line.
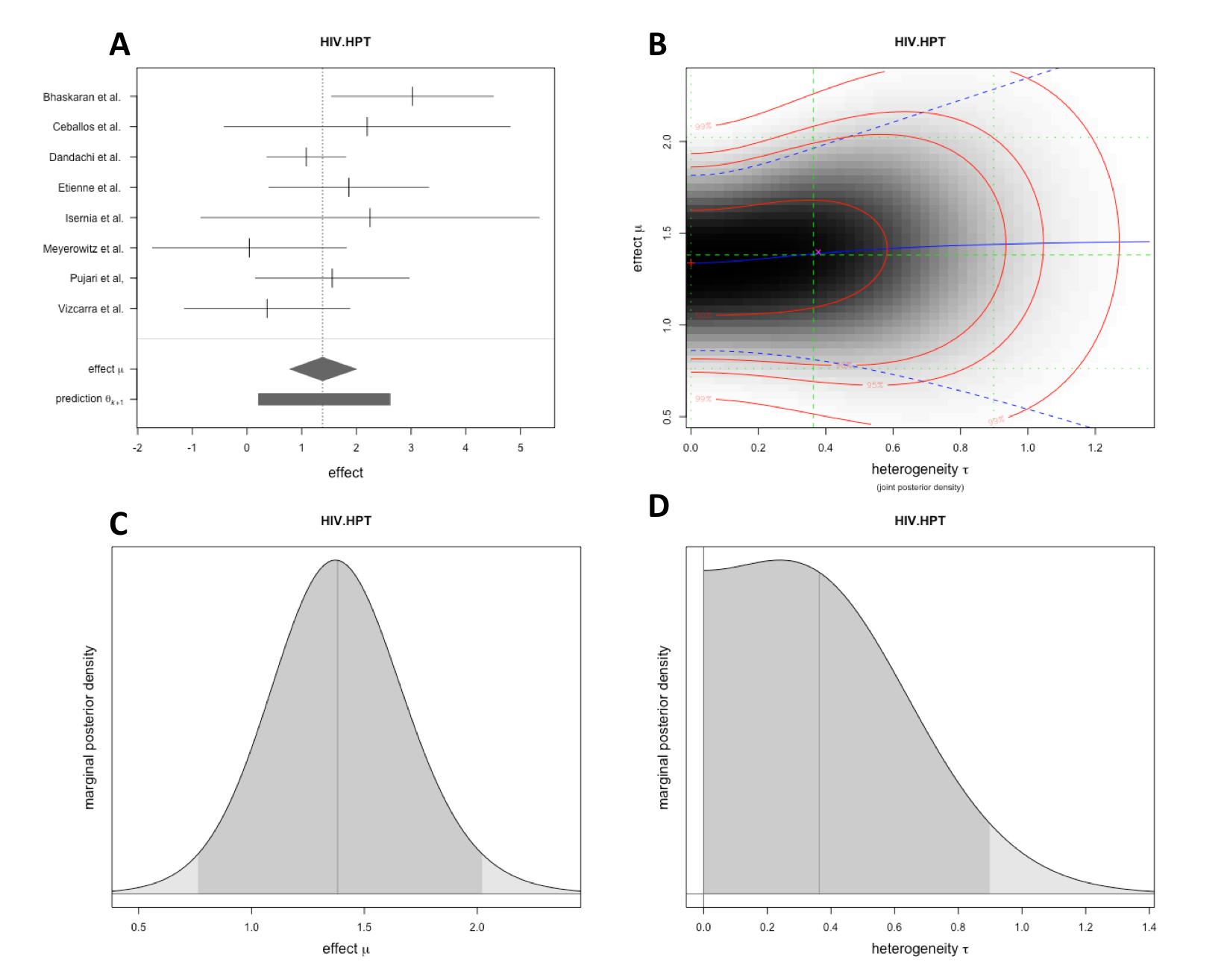


**Figure S8 Posterior distribution of the fixed effects and random effects τ for the association of HIV/Cardiovascular disease in comparison to HIV monoinfection with severe COVID-19 outcomes with a Bayesian approach with half normal distribution prior (scale of 0.5).**

(A) is a simple forest plot showing estimates and 95% intervals illustrating the input data along with the estimated mean eﬀect µ and a prediction interval for the eﬀect θ_k+1_ in a future study. (B) illustrates the joint posterior density of heterogeneity τ and eﬀect µ, with darker shading corresponding to higher probability density. The red lines indicate (approximate) 2-dimensional credible regions, and the green lines show marginal posterior medians and 95% credible intervals. The blue lines show the conditional posterior mean eﬀect µ(τ) as a function of the heterogeneity τ along with a 95% interval based on its conditional standard error σ (τ). The red cross (+) indicates the posterior mode, while the pink cross (×) shows the ML estimate. The two bottom plots (C) and (D) show the marginal posterior densities of eﬀect µ and heterogeneity τ. 95% credible intervals are indicated with a darker shading, and the posterior median is shown by a vertical line.


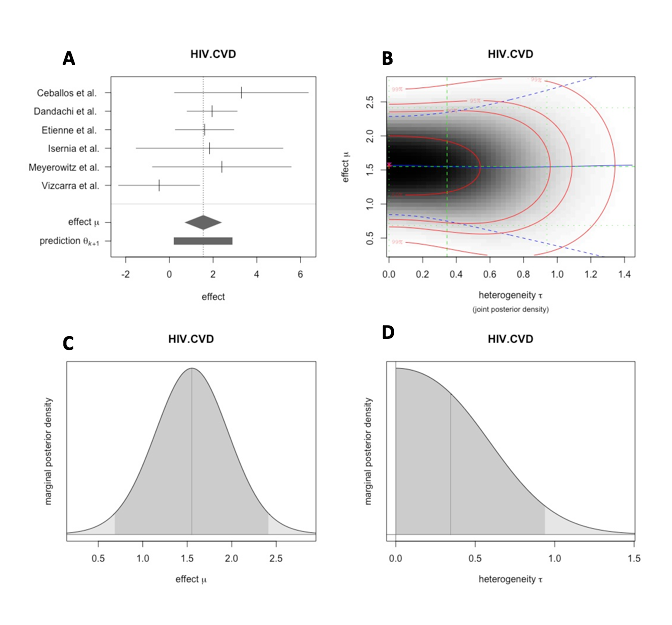


**Figure S9 Posterior distribution of the fixed effects and random effects τ for the association of HIV/Respiratory disease in comparison to HIV monoinfection with severe COVID-19 outcomes with a Bayesian approach with half normal distribution prior (scale of 0.5).**

(A) is a simple forest plot showing estimates and 95% intervals illustrating the input data along with the estimated mean eﬀect µ and a prediction interval for the eﬀect θ_k+1_ in a future study. (B) illustrates the joint posterior density of heterogeneity τ and eﬀect µ, with darker shading corresponding to higher probability density. The red lines indicate (approximate) 2-dimensional credible regions, and the green lines show marginal posterior medians and 95% credible intervals. The blue lines show the conditional posterior mean eﬀect µ(τ) as a function of the heterogeneity τ along with a 95% interval based on its conditional standard error σ (τ). The red cross (+) indicates the posterior mode, while the pink cross (×) shows the ML estimate. The two bottom plots (C) and (D) show the marginal posterior densities of eﬀect µ and heterogeneity τ. 95% credible intervals are indicated with a darker shading, and the posterior median is shown by a vertical line.


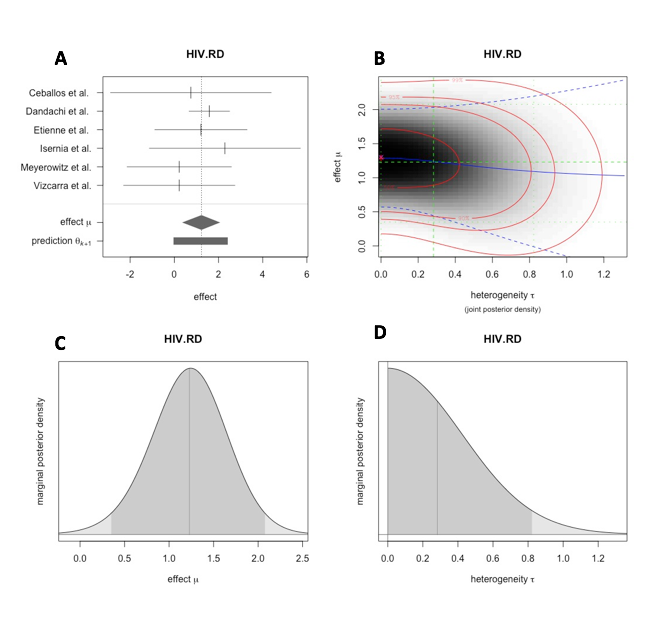


**Figure S10 Posterior distribution of the fixed effects and random effects τ for the association of HIV/Chronic kidney disease in comparison to HIV monoinfection with severe COVID-19 outcomes with a Bayesian approach with half normal distribution prior (scale of 0.5).**

(A) is a simple forest plot showing estimates and 95% intervals illustrating the input data along with the estimated mean eﬀect µ and a prediction interval for the eﬀect θ_k+1_ in a future study. (B) illustrates the joint posterior density of heterogeneity τ and eﬀect µ, with darker shading corresponding to higher probability density. The red lines indicate (approximate) 2-dimensional credible regions, and the green lines show marginal posterior medians and 95% credible intervals. The blue lines show the conditional posterior mean eﬀect µ(τ) as a function of the heterogeneity τ along with a 95% interval based on its conditional standard error σ (τ). The red cross (+) indicates the posterior mode, while the pink cross (×) shows the ML estimate. The two bottom plots (C) and (D) show the marginal posterior densities of eﬀect µ and heterogeneity τ. 95% credible intervals are indicated with a darker shading, and the posterior median is shown by a vertical line.


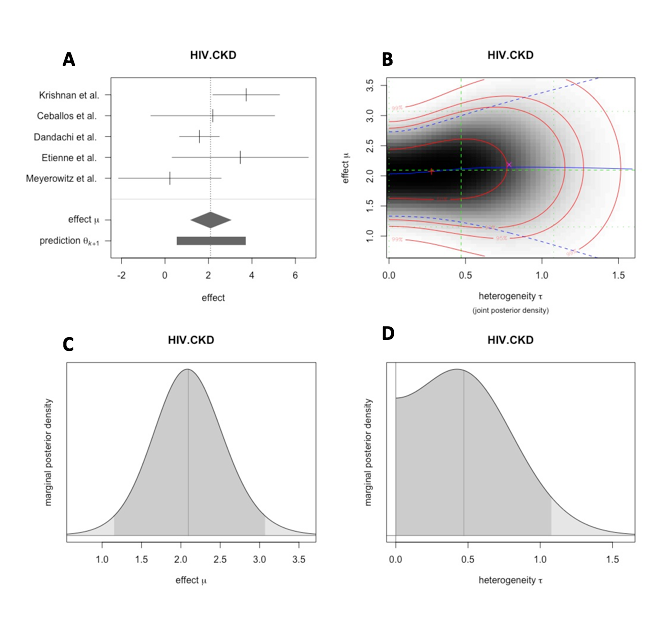


**Supplementary References**

1. Bhaskaran K, Rentsch CT, MacKenna B, Schultze A, Mehrkar A, Bates CJ, et al. HIV infection and COVID-19 death: a population-based cohort analysis of UK primary care data and linked national death registrations within the OpenSAFELY platform. The lancet HIV. 2021;8(1):e24-e32.

2. Boulle A, Davies M-A, Hussey H, Ismail M, Morden E, Vundle Z, et al. Risk factors for COVID-19 death in a population cohort study from the Western Cape Province, South Africa. Clinical infectious diseases : an official publication of the Infectious Diseases Society of America. 2020.

3. Ceballos ME, Ross P, Lasso M, Dominguez I, Puente M, Valenzuela P, et al. Clinical characteristics and outcomes of people living with HIV hospitalized with COVID-19: a nationwide experience. Int J STD AIDS. 2021:956462420973106.

4. Dandachi D, Geiger G, Montgomery MW, Karmen-Tuohy S, Golzy M, Antar AAR, et al. Characteristics, Comorbidities, and Outcomes in a Multicenter Registry of Patients with HIV and Coronavirus Disease-19. Clinical infectious diseases : an official publication of the Infectious Diseases Society of America. 2020.

5. Etienne N, Karmochkine M, Slama L, Pavie J, Batisse D, Usubillaga R, et al. HIV infection and COVID-19: risk factors for severe disease. Aids. 2020;34(12):1771-4.

6. Isernia V, Julia Z, Le Gac S, Bachelard A, Landman R, Lariven S, et al. SARS-COV2 infection in 30 HIV-infected patients followed-up in a French University Hospital. International journal of infectious diseases : IJID : official publication of the International Society for Infectious Diseases. 2020;101:49-51.

7. Meyerowitz EA, Kim AY, Ard KL, Basgoz N, Chu JT, Hurtado RM, et al. Disproportionate burden of coronavirus disease 2019 among racial minorities and those in congregate settings among a large cohort of people with HIV. AIDS (London, England). 2020;34(12):1781-7.

8. Pujari S, Gaikwad S, Chitalikar A, Dabhade D, Joshi K, Bele V. Coronavirus Disease 19 Among People Living with HIV in Western India: An Observational Cohort Study. AIDS Res Hum Retroviruses. 2021.

9. Vizcarra P, Perez Elias MJ, Quereda C, Moreno A, Vivancos M, Dronda F, et al. Description of COVID-19 in HIV-infected individuals: a single-centre, prospective cohort. The Lancet HIV. 2020;7.
